# Diagnostic accuracy of circulating microRNAs for hepatitis C virus-associated hepatocellular carcinoma: a systematic review and meta-analysis

**DOI:** 10.1186/s12879-022-07292-8

**Published:** 2022-04-01

**Authors:** Yicheng Huang, Yingsha Chen, Sheng Tu, Jiajie Zhang, Yunqing Qiu, Wei Yu

**Affiliations:** 1 Center for General Practice Medicine, Department of Infectious Diseases, Zhejiang Provincial People’s Hospital (Affiliated People’s Hospital, Hangzhou Medical College), Hangzhou, China; 2 State Key Laboratory for Diagnosis and Treatment of Infectious Diseases, National Clinical Research Center for Infectious Diseases, Collaborative Innovation Center for Diagnosis and Treatment of Infectious Diseases, The First Affiliated Hospital, Zhejiang University School of Medicine, Hangzhou, China

**Keywords:** Hepatitis C virus, Hepatocellular carcinoma, microRNAs, Biomarkers

## Abstract

**Aims:**

The purpose of this study was to perform an assessment of circulating microRNAs (miRNAs) as promising biomarker for hepatitis C virus (HCV)-associated hepatocellular carcinoma (HCV-HCC) through a meta-analysis.

**Methods:**

A comprehensive literatures search extended up to March 1, 2020 in PubMed, Cochrane library, Embase, Web of Science, Scopus and Ovid databases. The collected data were analyzed by random-effects model, the pooled sensitivity (SEN), specificity (SPE), positive and negative likelihood ratios (PLR and NLR), diagnostic odds ratio (DOR), and area under the curve (AUC) were used to explore the diagnostic performance of circulating miRNAs. Meta-regression and subgroup analysis were further carried out to explore the heterogeneity.

**Results:**

A total of 16 articles including 3606 HCV-HCC patients and 3387 HCV patients without HCC were collected. The pooled estimates indicated miRNAs could distinguish HCC patients from chronic hepatitis C (CHC) and HCV-associated liver cirrhosis (HCV-LC), with a SEN of 0.83 (95% CI, 0.79–0.87), a SPE of 0.77 (95% CI, 0.71–0.82), a DOR of 17 (95% CI, 12–28), and an AUC of 0.87 (95% CI, 0.84–0.90). The combination of miRNAs and AFP showed a better diagnostic accuracy than each alone. Subgroup analysis demonstrated that diagnostic accuracy of miRNAs was better for plasma types, up-regulated miRNAs, and miRNA clusters. There was no evidence of publication bias in Deeks’ funnel plot.

**Conclusions:**

Circulating miRNAs, especially for miRNA clusters, have a relatively high diagnostic value for HCV-HCC from CHC and HCV-LC.

**Supplementary Information:**

The online version contains supplementary material available at 10.1186/s12879-022-07292-8.

## Background

Hepatitis C virus (HCV) infection is one of the main risk factors for hepatocellular carcinoma (HCC) development. Approximately 399,000 people are estimated to die annually from HCV-associated liver cirrhosis (HCV-LC) and HCC [1]. The rate of progression from chronic hepatitis C (CHC) to HCC is variable and the cancerogenesis mechanism of HCV has yet completely known [2]. In addition, the only strategy to implement for HCV-HCC is still prevention despite advances in an era of all-oral direct-acting antivirals (DAAs) regimens [3]. However, detection of early HCC remains difficult due to technical challenge in non-invasive methods [4]. Therefore, new biomarkers with higher diagnostic accuracy are mandatory for early HCV-associated HCC (HCV-HCC).

MicroRNAs (miRNAs) could regulate gene expression and control cellular processes [5]. Numerous studies indicate that dysregulation of miRNAs expression lead to pathological processes of several types of cancer [6]. Recently, it has been increasingly recognized the meaningful properties of circulating miRNAs as the potential biomarkers for HCC [7]. Several HCV-HCC related miRNAs, such as miR-16, miR-122, miR-150, miR-182, miR-199a, miR-211, and miR-224, have been confirmed [8–11]. However, no consensus on diagnosis accuracy of circulating miRNAs for HCV-HCC has yet emerged. In the present study, a systematic review and meta-analysis was performed to evaluate the expression levels of circulating miRNAs of patients with HCV infections, in order to clarify the diagnostic accuracy of HCC from CHC and HCV-LC.

## Methods

### Search strategy and literatures selection

According to the guidelines of diagnostic meta-analysis, a systematic search of the literatures was performed by two investigators (WY and YCH) using the sources of Pubmed, Cochrane library, Embase, Web of Science, Scopus and Ovid from inception through the end of March 1, 2020. The retrieval terms included: "Liver Neoplasms" or "Hepatic Neoplasms" or “Liver Cancers” or "Carcinoma, Hepatocellular" or "Liver Cell Carcinoma" and "Hepatitis C" and "microRNAs" or "miRNA".

Literatures included according to the following information: (1) both HCC groups and control groups ware HCV-related; (2) the detection of the circulating miRNAs was related to HCV-HCC; (3) true positive (TP), true negative (TN), false positive (FP), and false negative (FN) of the miRNAs were reported or could be calculated. On the other hand, the exclusion criteria were shown as following: (1) meta-analysis, case reports, reviews or letters; (2) repetitive research; (3) the obtained miRNAs were not from blood; (4) insufficient data were not available for the diagnosis value.

### Data collection and quality assessment

The final set of the included studies was assessed by two investigators (YSC and ST). The final judgment originated from any disagreements were made by a third investigator (YCH). The data of included studies were extracted including the name of first author, publication year, ethnicity, the type and alteration of circulating miRNAs, sample source, normalization controls, alpha-fetoprotein (AFP), numbers of HCC, CHC and HCV-LC, and numbers of TP, TN, FP, FN observations.

The quality of included studies was assessed using Quality Diagnostic Accuracy Studies-2 (QUADAS-2) criteria by two independent authors (WY and JJZ) [12]. The disagreement was settled by a third reviewer (YCH).

### Data synthesis and analysis

All the statistical analysis was conducted by STATA version 14 (STATA Corp, College Station, TX, USA). The pooled sensitivity (SEN), specificity (SPE), positive and negative likelihood ratios (PLR and NLR), diagnostic odds ratio (DOR), summary receiver operating characteristic (SROC) curve and area under the curve (AUC) were calculated for circulating miRNAs using bivariate random-effects regression model. In addition, potential sources of heterogeneity were explored using threshold effect analysis and regression analysis. Then subgroup analysis was further analyzed based on varied factors. Moreover, differences between the overall accuracy (OA) of miRNAs, AFP or the combination of miRNAs and AFP in discriminating HCV-HCC patients from controls were analyzed using SPSS Statistics 20 (IBM, China). Publication bias were assessed by Deeks’ funnel plot. *P*-value less than 0.05 was considered statistically significant.

## Results

### Included studies

**Fig. 1:**
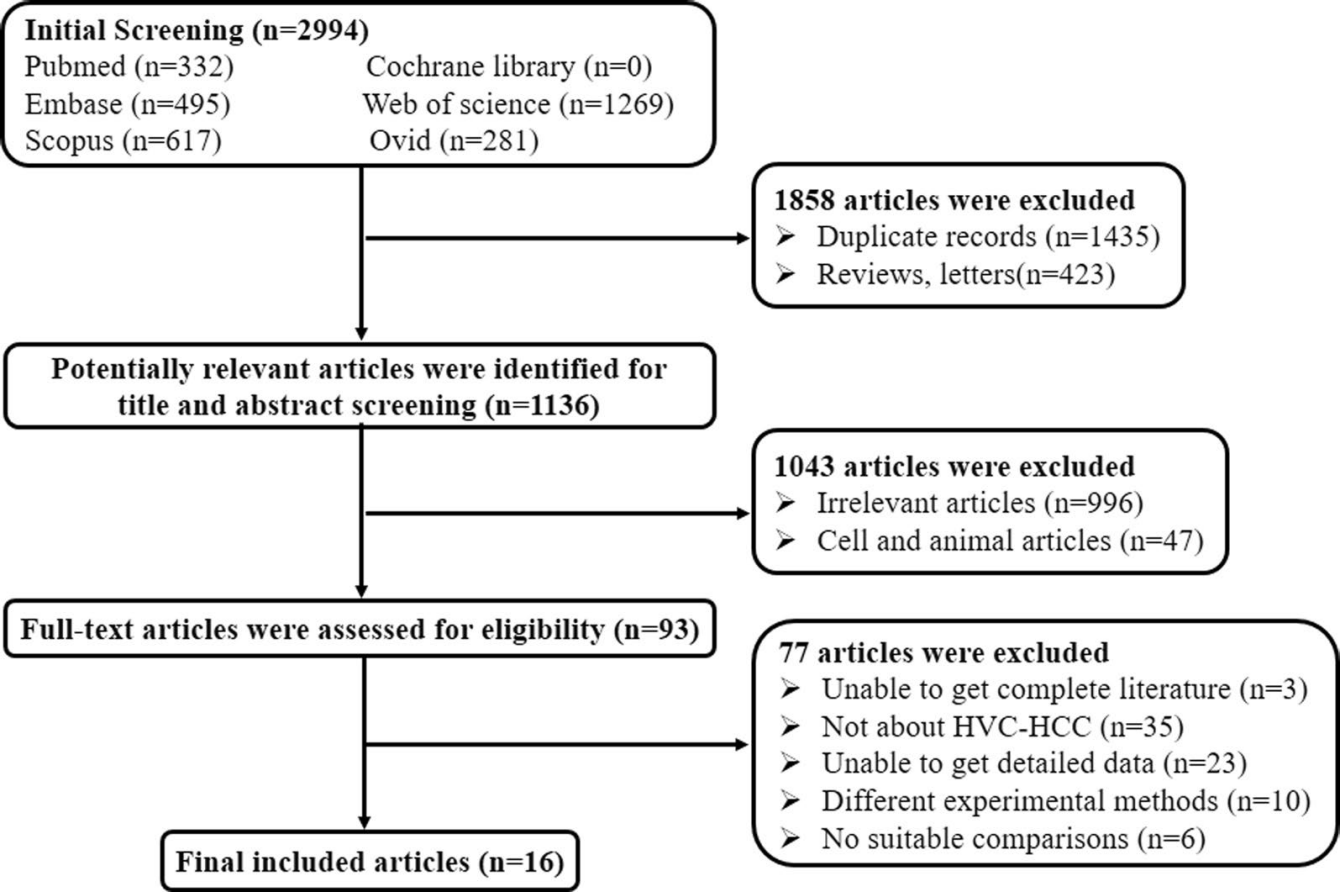
Flowchart of literatures selection in this meta-analysis

The process of studies selection was shown in Fig. 1. A total of 2994 articles were identified from initial literatures search, including 332 in Pubmed, 495 in Embase, 1269 in Web of Science, and 617 in Scopus and 281 in Ovid. After preliminary selection, 1858 articles were removed due to duplicate records and unfit literary forms. Finally, 16 articles were included according to inclusion and exclusion criteria [9–11, 13–25].

Among 16 articles, we extracted 39 studies including 3607 HCV-HCC patients and 3387 HCV infected patients as control population. The characteristics of included studies were shown in Table 1. Quantitative real-time reverse transcription PCR (qRT-PCR) was used to measure the expression of miRNAs from 34 serum specimens and 5 plasma specimens. Among 39 studies, 4 studies assessed multiple miRNAs for HCV-HCC diagnosis, and the other 35 studies were focused on single miRNA. The conduct of patient selection introduced unclear risk in 8 articles during quality assessment [10, 13–17, 19, 24] (Additional file 1: Fig. S1).

**Table 1:** Characteristics of studies included in this meta-analysis

First author	Year	Region	MicroRNAs	Regulation mode	Specimen	Internal reference types in qRT-PCR	Sample size				Diagnostic power			Ref.
							Case	Number	Control	Number	SEN (%)	SPE (%)	AUC	
El-Garem H	2014	Egypt	miR-211	Downregulated	Serum	SNORD68	HCC	30	LC + HC	60	87	40	0.655	[9]
El-Abd NE	2015	Egypt	miR-16	Downregulated	Serum	RNU48	HCC	40	CHC	40	57.5	70	0.638	[10]
Motawi TK	2015	Egypt	miR-19a	Downregulated	Serum	SNORD68	HCC	112	LC	50	85.7	75	0.816	[13]
Motawi TK	2015	Egypt	miR-296	Upregulated	Serum	SNORD68	HCC	112	LC	50	76.9	56.5	0.666	[13]
Motawi TK	2015	Egypt	miR-195	Downregulated	Serum	SNORD68	HCC	112	LC	50	66.7	70.8	0.665	[13]
Motawi TK	2015	Egypt	miR-192	Upregulated	Serum	SNORD68	HCC	112	LC	50	78.6	62.5	0.730	[13]
Motawi TK	2015	Egypt	miR-34a	Upregulated	Serum	SNORD68	HCC	112	LC	50	51.9	82.6	0.663	[13]
Motawi TK	2015	Egypt	miR-146a	Upregulated	Serum	SNORD68	HCC	112	LC	50	96.4	73.9	0.880	[13]
Amr KS	2017	Egypt	miR-122	Downregulated	Plasma	RUN6B	HCC	40	CHC	40	87.5	97.5	NA	[11]
Amr KS	2017	Egypt	miR-244	Upregulated	Plasma	RUN6B	HCC	40	CHC	40	87.5	97	NA	[11]
Shaker O	2017	Egypt	miR-101-1	Downregulated	Serum	SNORD68	HCC	37	LC + HC	78	73	71	0.763	[14]
Shaker O	2017	Egypt	miR-221	Upregulated	Serum	SNORD68	HCC	37	LC + HC	78	56.8	73.9	0.673	[14]
Elemeery MN	2017	Egypt	miR-214-5P	Downregulated	Serum	SNORD68	HCC	224	CHC	250	92.2	75.5	0.842	[15]
Elemeery MN	2017	Egypt	miR-494	Upregulated	Serum	SNORD68	HCC	224	CHC	250	77	56	0.631	[15]
Elemeery MN	2017	Egypt	miR-138b	Downregulated	Serum	SNORD68	HCC	224	CHC	250	68.2	58.2	0.642	[15]
Elemeery MN	2017	Egypt	miR-125b	Downregulated	Serum	SNORD68	HCC	224	CHC	250	92.6	55.4	0.769	[15]
Elemeery MN	2017	Egypt	miR-1269	Upregulated	Serum	SNORD68	HCC	224	CHC	250	78.6	59.8	0.691	[15]
Elemeery MN	2017	Egypt	miR-145	Downregulated	Serum	SNORD68	HCC	224	CHC	250	81.5	51.5	0.624	[15]
Elemeery MN	2017	Egypt	miR-375	Downregulated	Serum	SNORD68	HCC	224	CHC	250	96.4	69.3	0.811	[15]
Elemeery MN	2017	Egypt	miRNA clusters(4)	NA	Serum	SNORD68	HCC	224	Fibrosis	150	96,7	94.3	0.945	[15]
Elemeery MN	2017	Egypt	miRNA clusters(4)	NA	Serum	SNORD68	HCC	224	Fibrosis	100	98.7	98.3	0.990	[15]
Shaheen NMH	2018	Egypt	miR-182	Downregulated	Serum	Cel-miR-39	HCC	40	CHC	20	72.5	65	0.675	[16]
Shaheen NMH	2018	Egypt	miR-150	Downregulated	Serum	Cel-miR-39	HCC	40	CHC	20	67.5	70	0.704	[16]
Rashad NM	2018	Egypt	miR-27a	Upregulated	Serum	SNORD68	HCC	51	LC	39	96.7	71.7	0.897	[17]
Rashad NM	2018	Egypt	miR-18b	Upregulated	Serum	SNORD68	HCC	51	LC	39	75.6	46.7	0.732	[17]
Rashad NM	2018	Egypt	miRNA clusters(2)	Upregulated	Serum	SNORD68	HCC	51	LC	39	91.1	71.7	0.821	[17]
El-Hamouly MS	2019	Egypt	miR-301	Upregulated	Plasma	U6	HCC	42	CHC	48	78.6	89.6	0.890	[18]
Ali LH	2019	Egypt	miR-215	Upregulated	Serum	SNORD68	HCC	60	LC	60	97.1	91	0.997	[19]
Shehab-Eldeen S	2019	Egypt	miR-122	Downregulated	Plasma	U6	HCC	20	CHC	20	95	81	0.930	[20]
Shehab-Eldeen S	2019	Egypt	miR-224	Upregulated	Plasma	U6	HCC	20	CHC	20	85	79	0.770	[20]
Sun Q	2019	China	miR-331-3p	Upregulated	Serum	Cel-miR-39	HCC	40	CHC	106	62.5	74.5	0.748	[21]
Sun Q	2019	China	miR-23b-3p	Downregulated	Serum	Cel-miR-39	HCC	40	CHC	106	85.8	65	0.806	[21]
Sun Q	2019	China	miR-331-3p	Upregulated	Serum	Cel-miR-39	HCC	40	LC	47	75	85.1	0.832	[21]
Sun Q	2019	China	miR-23b-3p	Downregulated	Serum	Cel-miR-39	HCC	40	LC	47	85.1	65	0.796	[21]
Oura K	2019	Japan	miR-125a-5p	Upregulated	Serum	Cel-miR-39	HCC	20	LC + CHC	20	80	100	0.980	[22]
Weis A	2019	Australia	miRNA clusters(3)	NA	Serum	SNORD68	HCC	20	CHC	20	80	95	0.940	[23]
Fatma A	2019	Egypt	miR-19a	Upregulated	Serum	SNORD68	HCC	40	LC + CHC	40	60	67.5	0.616	[24]
Fatma A	2019	Egypt	miR-223	Downregulated	Serum	SNORD68	HCC	40	LC + CHC	40	60	95	0.816	[24]
Aly DM	2020	Egypt	miR-let-7a-1	Downregulated	Serum	SNORD68	HCC	40	LC	20	70	82.5	0.740	[25]

*qRT-PCR* quantitative real-time reverse transcription PCR, *HCC* hepatocellular carcinoma, *LC* liver cirrhosis, *CHC* chronic hepatitis C, *SEN* sensitivity, *SPE* specificity, *AUC* area under the curve, Ref., references

### Accurate diagnosis of miRNAs compared with AFP in HCV-HCC patients

The threshold effect was evaluated before data combination. The correlation coefficient was 0.33 (P = 0.11), indicating no significant threshold effect in the present study.

**Fig. 2:**
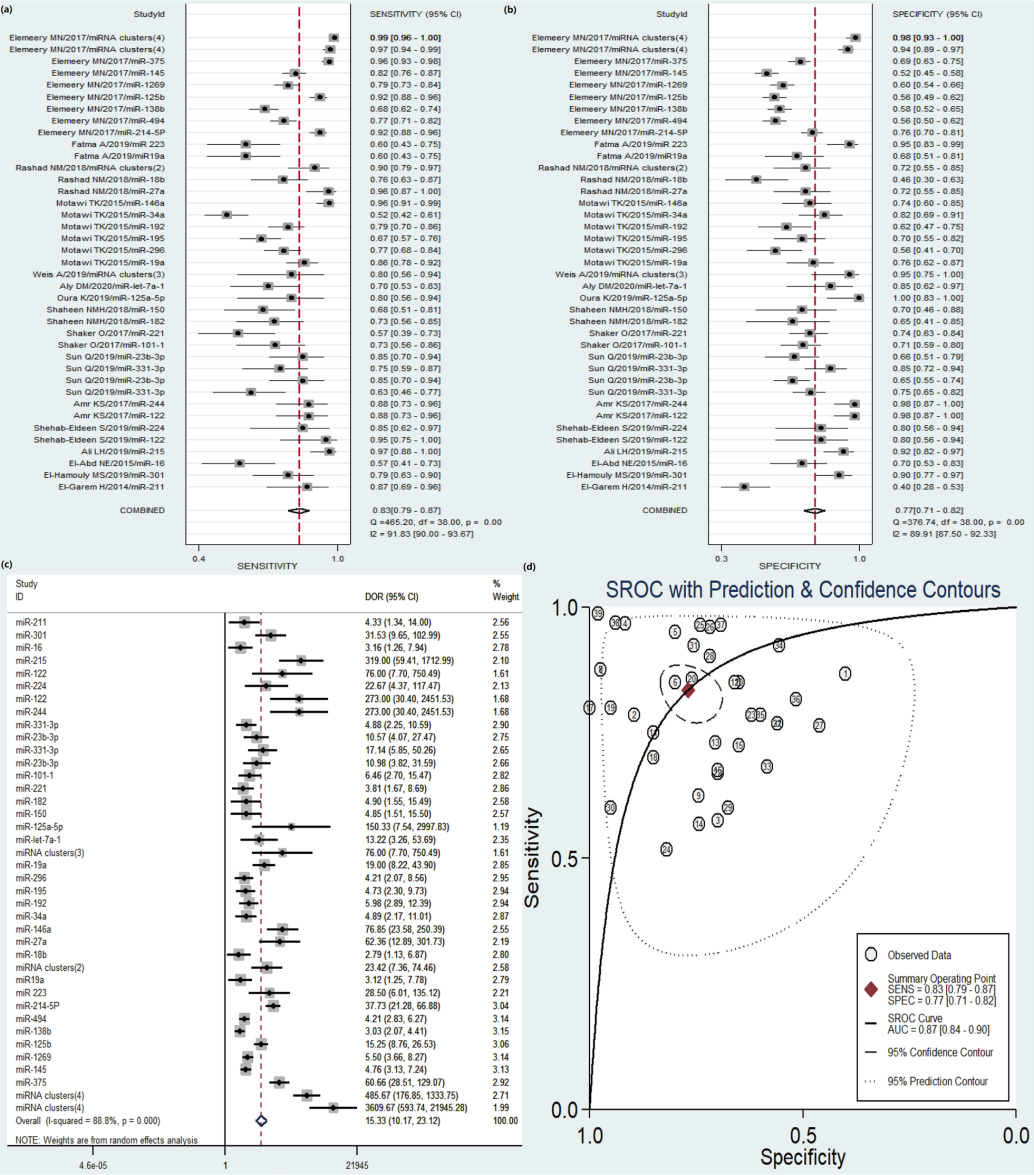
Forest plots of pooled sensitivity (SEN), specificity (SPE), diagnostic odds ratio (DOR), and summary receiver operating characteristic (SROC) curve of circulating miRNAs for diagnosis of HCV-HCC among 39 studies. **a** SEN; **b** SPE; **c** DOR; **d** SROC curve

Significant heterogeneity was observed among 39 studies (I-squared = 91.83% for SEN, I-squared = 89.91% for SPE, I-squared = 88.8% for DOR, respectively), therefore, random-effects model was selected for the overall analysis. Forest plots of SEN, SPE and DOR results were shown in Fig. 2a–c. The overall pooled results were summarized as following: SEN 0.83 (95% CI, 0.79–0.87), SPE 0.77 (95% CI, 0.71–0.82), PLR 3.6 (95% CI, 2.8–4.7), NLR 0.21 (95% CI, 0.16–0.29), and DOR 17 (95% CI, 12–28) (Additional file 1: Table S1). The AUC value was 0.87 (95% CI, 0.84–0.90) in the overall SROC curve (Fig. 2d). The above results manifested the diagnostic accuracy of circulating miRNAs for HCC is relatively high.

**Fig. 3:**
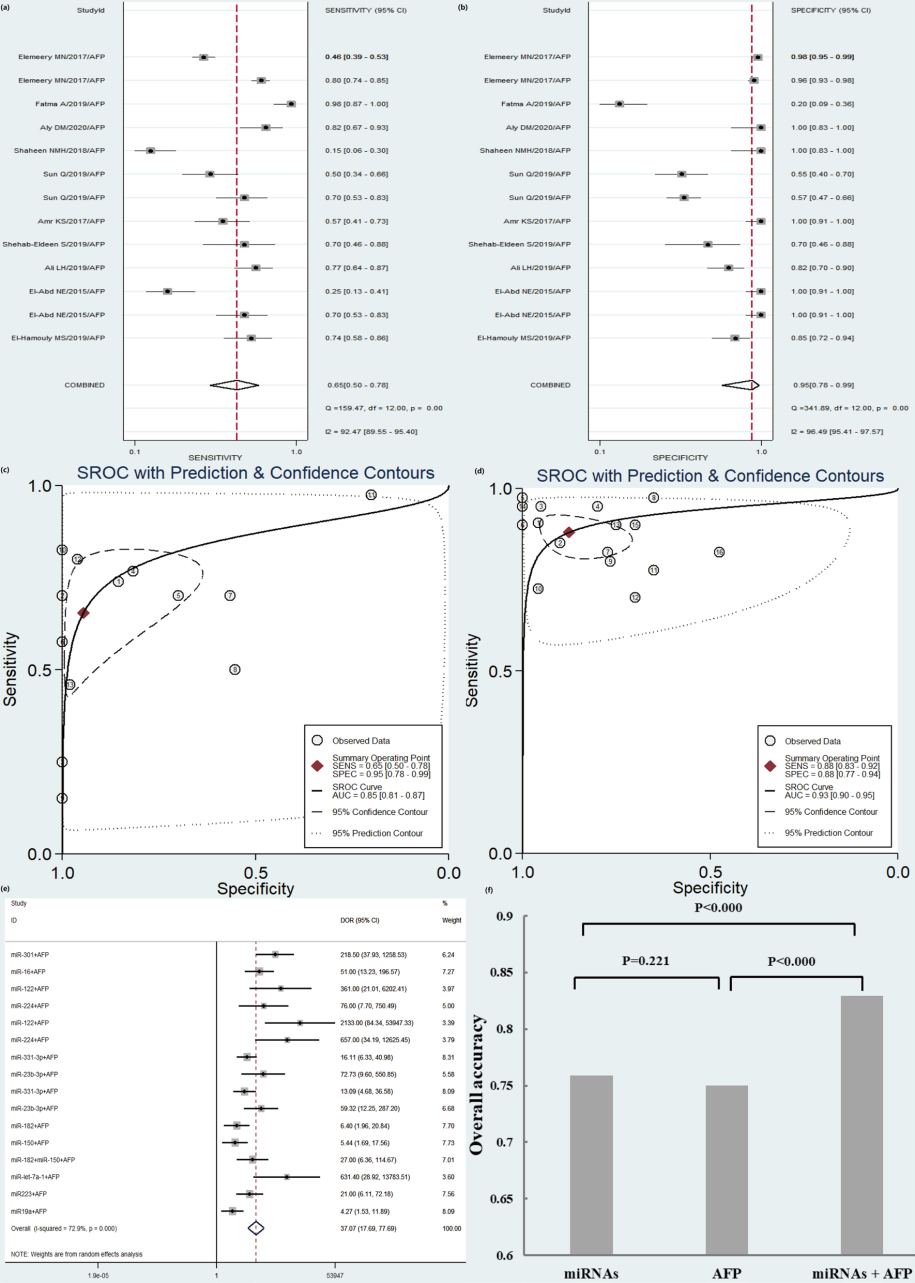
Diagnostic accuracy of AFP for HCV-HCC diagnosis compared with circulating miRNAs combined with AFP. **a** sensitivity (SEN) of AFP; **b** specificity (SPE) of AFP; **c** SROC of AFP; **d** summary receiver operating characteristic (SROC) curve of miRNAs combined with AFP; **e** diagnostic odds ratio (DOR) of circulating miRNAs combined with AFP; **f** overall accuracy (OA) value

Thirteen studies determined the accuracy of AFP diagnosis, and 16 studies determined the combination of miRNAs and AFP for HCV-HCC patients. miRNAs combined with AFP showed a higher accuracy than AFP alone with SEN of 0.88 versus 0.65, SPE of 0.88 versus 0.95, PLR of 7.1 versus 12.0, NLR of 0.14 versus 0.37, DOR of 51 versus 33, and AUC of 0.93 versus 0.85, respectively (Fig. 3a–c and Additional file 1: Table S1). The OA value analysis indicated that the combination of miRNAs and AFP had a significantly higher accuracy for HCV-HCC than AFP or miRNAs alone (*P* < 0.000). Although the DOR of AFP is higher than miRNAs alone (33 versus 17), there was no significant difference existed in the diagnostic accuracy of the OA value between the two methods (Fig. 3d–f).

### Meta-regression analysis to exploring Sources of Heterogeneity

Meta-regression analysis was used to explore sources of heterogeneity. Region, specimen types, regulation mode, internal reference types, miRNAs profiling, sample size, control groups were internal considered as parameters (Table 2). It can be seen from the results that the specimen types (P = 0.03), regulation mode (P = 0.01), miRNAs profiling (P < 0.01) had statistical significance. However, the parameter region (P = 0.07), internal reference types (P = 0.09), sample size (P = 0.12) and control groups (P = 0.14) were not statistically significant (P > 0.05).

**Table 2:** The meta-regression analysis of variable parameters

Parameters	Sensitivity				Specificity				Joint model		
	estimate (95% CI)	Coef	Z	P >|z|	estimate (95% CI)	Coef	Z	P >|z|	I-squared (95% CI)	LRTChi	P value
Region	0.75 (0.45–0.92)	1.09	− 0.77	0.44	0.92 (0.74–0.98)	2.43	1.66	0.10	61.57 (13.37–100.00)	5.2	0.07
Specimen types	0.88 (0.71–0.96)	2.00	0.77	0.51	0.93 (0.82–0.97)	2.57	2.47	0.01	72.39 (38.81–100.00)	7.24	0.03
Regulation mode	0.88 (0.81–0.93)	2.02	1.17	0.24	0.87 (0.79–0.92)	1.90	2.21	0.03	77.68 (51.46–100.00)	8.96	0.01
Internal reference types	0.78 (0.63–0.89)	1.28	− 0.77	0.44	0.85 (0.74–0.92)	1.77	1.35	0.18	59.09 (7.72–100.00)	4.89	0.09
miRNAs profiling	0.96 (0.89–0.98)	3.11	2.70	0.01	0.94 (0.84–0.98)	2.70	2.73	0.01	85.14 (69.07–100.00)	13.46	< 0.01
Sample size	0.88 (0.78–0.93)	1.95	0.90	0.37	0.69 (0.53–0.81)	0.80	− 1.33	0.26	52.19 (0.00–100.00)	4.18	0.12
Control groups	0.86 (0.79–0.91)	1.78	0.54	0.59	0.82 (0.75–0.88)	1.54	1.25	0.21	49.72 (0.00–100.00)	3.98	0.14

*miRNAs* microRNAs, *95% CI* 95% confidence intervals, *Coef.* coefficient

### Subgroup analyses

Subgroup analyses were performed based on region, specimen types, regulation mode, internal reference types, miRNAs profiling, sample size, source of control. Majority of the research populations were Egypt (33 studies contained 3407 HCV-HCC patients and 3041 controls) with the pooled SEN of 0.84 (95% CI 0.79–0.89), SPE of 0.76 (95% CI 0.69–0.82), PLR of 3.5 (95% CI 2.6–4.6), NLR of 0.21 (95% CI 0.15–0.30), DOR 17 (95% CI 9–30) of and AUC of 0.87 (95% CI 0.84–0.90). The difference among subgroup analysis based on internal reference types, miRNAs profiling, and sample size was minimal (Table 3).

**Table 3:** Summary diagnostic power based on subgroup analyses

Subgroup	Number of studies	Number of HCC	Number of controls	SEN (95% CI)	I-squared (%)	SPE (95% CI)	I-squared (%)	PLR (95% CI)	NLR (95% CI)	DOR (95% CI)	AUC (95% CI)
*Region*											
Africa	33	3407	3041	0.84 (0.79–0.89)	92.93	0.76 (0.69–0.82)	90.56	3.5 (2.6–4.6)	0.21 (0.15–0.30)	17 (9–30)	0.87 (0.84–0.90)
Asia	5	180	326	0.77 (0.68–0.85)	49.68	0.78 (0.63–0.88)	75.02	3.5 (2.0–5.9)	0.29 (0.20–0.42)	12 (6–26)	0.84 (0.80–0.87)
*Specimen types*											
Serum	34	3445	3219	0.83 (0.77–0.87)	92.28	0.74 (0.68–0.79)	88.95	3.2 (2.5–4.1)	0.23 (0.17–0.32)	14 (8–23)	0.85 (0.82–0.88)
Plasm	5	162	168	0.86 (0.79–0.90)	0	0.91 (0.83–0.96)	62.13	10.0 (4.7–21.2)	0.16 (0.11–0.23)	64 (25–164)	0.87 (0.84–0.90)
*Regulation mode*											
Upregulated	18	1388	1276	0.81 (0.74–0.87)	89.46	0.77 (0.68–0.83)	87.71	3.5 (2.5–4.9)	0.25 (0.17–0.36)	14 (7–27)	0.86 (0.82–0.89)
Downregulated	18	1751	1841	0.82 (0.75–0.87)	89.88	0.71 (0.63–0.78)	84.56	2.8 (2.2–3.6)	0.25 (0.18,0.35)	11 (7–18)	0.83 (0.80–0.86)
*Internal reference types*											
SNORD 68	26	3145	2813	0.85 (0.79–0.90)	93.97	0.74 (0.66–0.80)	91.15	3.2 (2.4–4.4)	0.20 (0.13–0.31)	16 (8–31)	0.86 (0.83–0.89)
non-SNORD 68	13	462	574	0.79 (0.72–0.84)	62.26	0.83 (0.74–0.90)	78.75	4.6 (2.8–7.6)	0.26 (0.19–0.35)	18 (8–39)	0.86 (0.83–0.89)
*miRNAs profiling*											
Single miRNA	35	3088	3078	0.77 (0.68–0.83)	89.68	0.74 (0.68–0.79)	85.88	3.1 (2.5–3.9)	0.26 (0.20–0.33)	12 (8–19)	0.84 (0.81–0.87)
miRNA clusters	4	519	309	0.95 (0.89–0.98)	85.29	0.92 (0.79–0.97)	88.77	11.8 (4.1–34.4)	0.05 (0.02–0.13)	237 (33–1687)	0.98 (0.97–0.99)
*Sample size*											
< 100	19	705	659	0.80 (0.74–0.85)	70.29	0.82 (0.72–0.88)	85.59	4.4 (2.8–6.8)	0.25 (0.19–0.33)	18 (9–33)	0.87 (0.83–0.89)
> 100	20	2902	2728	0.86 (0.78–0.91)	95.22	0.73 (0.65–0.80)	92.11	3.2 (2.3–4.4)	0.19 (0.12–0.32)	16 (7–36)	0.86 (0.82–0.89)
*Control groups*											
CHC	18	1950	2230	0.83 (0.77–0.88)	90.65	0.74 (0.66–0.82)	89.03	3.3 (2.3–4.5)	0.23 (0.16–0.32)	14 (8–26)	0.86 (0.83–0.89)
LC	13	1005	591	0.84 (0.75–0.90)	90.52	0.73 (0.66–0.79)	73.11	3.1 (2.4–4.2)	0.22 (0.14–0.36)	14 (7–28)	0.84 (0.80–0.87)

*miRNAs* microRNAs, *95% CI* 95% confidence intervals, *SEN* sensitivity, *SPE* specificity, *PLR* positive likelihood ratios, *NLR* negative likelihood ratios, *DOR* diagnostic odds ratio, *AUC* area under the curve, *HCC* hepatocellular carcinoma, *LC* liver cirrhosis, *CHC* chronic hepatitis C

The types of specimens could influence the diagnostic accuracy. miRNAs from plasma showed higher quality of detection than that from serum. The corresponding pooled SEN, SPE, PLR, NLR, DOR, and AUC were 0.86 versus 0.83, 0.91 versus 0.74, 10.0 versus 3.2, 0.16 versus 0.23, 64 versus 14, 0.87 versus 0.85, respectively. It is of note that the pooled SEN and DOR were significantly higher among multiple miRNAs subgroup compared with single miRNA (SEN 0.95 versus 0.81, DOR 237 versus 12), indicating significantly higher diagnostic accuracy of miRNA clusters for HCV-HCC.

There are 18 studies (2230 patients) of CHC and 13 studies of HCC-LC (591 patients) as controls. As shown in Table 3 and Additional file 1: Figs. S2, S3, the analysis based on source of control demonstrated the no significant difference of diagnostic accuracy between CHC and HCV-LC. However, among CHC group, miRNAs combined with AFP displayed a better diagnostic accuracy than miRNAs alone. The pooled results were displayed as following: SEN 0.89 (95% CI 0.83–0.93), SPE 0.88 (95% CI 0.76–0.95), PLR 7.7 (95% CI 3.5–17), NLR 0.12 (95% CI 0.07–0.20), DOR 63 (95% CI 20–203) and AUC 0.94 (95% CI 0.92–0.96).

### Publication bias and clinical utility of index

**Fig. 4:**
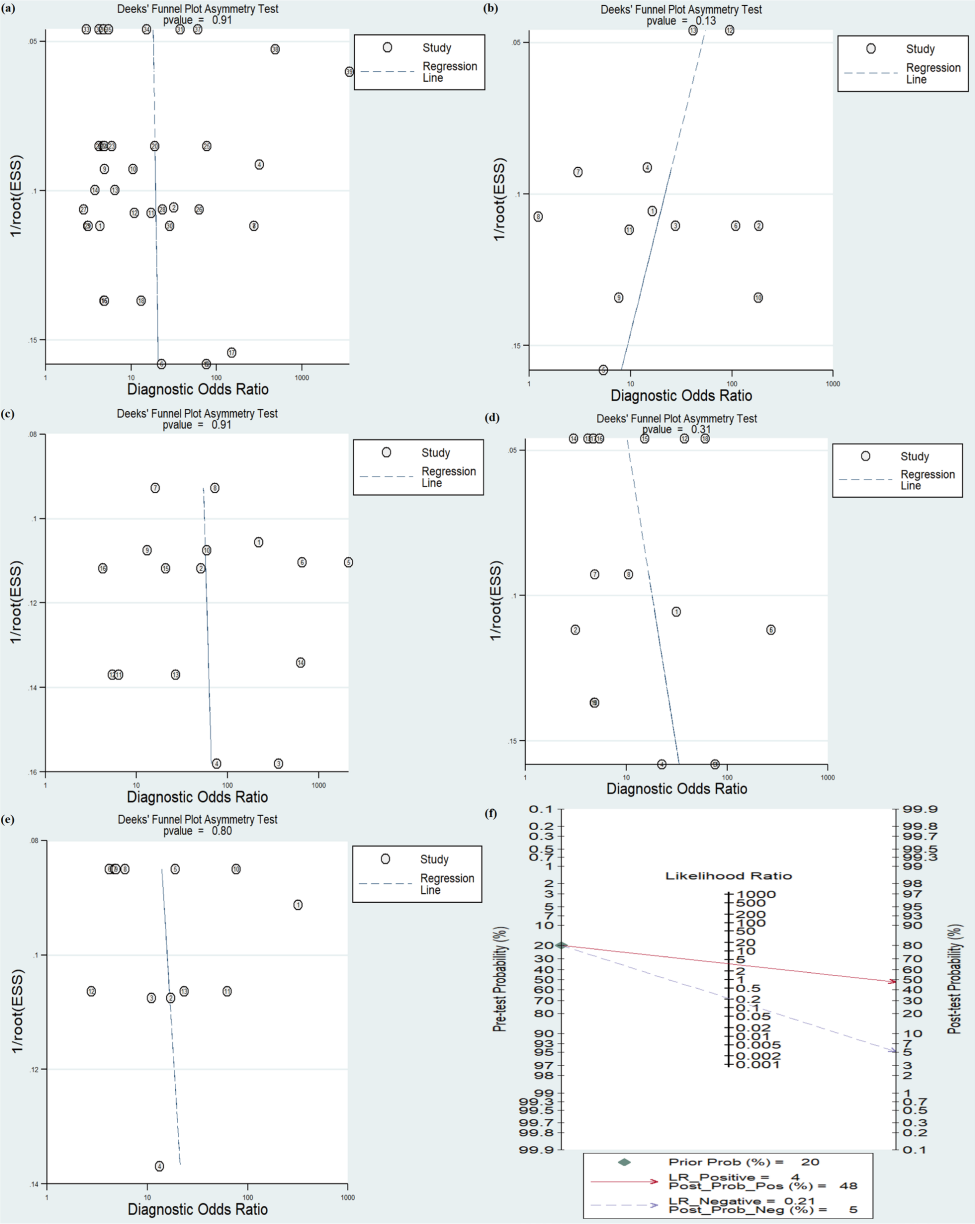
The Deeks’ funnel plot and Fagan’s Nomogram of the diagnostic meta-analysis. **a** Deeks’ funnel plot of miRNAs; **b** Deeks’ funnel plot of AFP; **c** Deeks’ funnel plot of miRNAs combined with AFP; **d** Deeks’ funnel plot of miRNAs for CHC subgroup; **e** Deeks’ funnel plot of miRNAs for HCV-LC subgroup; **f** Fagan’s Nomogram

Deeks’ funnel plot asymmetry test was conducted to investigate the publication bias of included studies. The P value for overall circulating miRNAs was 0.43, indicating little possibility of publication bias in our meta-analysis. In addition, P value of publication bias for AFP, miRNAs combined with AFP, CHC and HCV-LC were 0.13, 0.91, 0.31, and 0.80, respectively (Fig. 4a–e).

The post-test probabilities were assessed by Fagan’s Nomogram. When prior probability was 20%, post-test positive probability was 48% with PLR of 4 and negative probability was 5% with NLR of 0.21 (Fig. 4f).

## Discussion

DAA shows effective against HCV, however, direct evidence on the effects of antiviral therapy on HCV-HCC remains limited. Furthermore, the development of non-invasive markers for screening of HCC presents a challenge during the last decades. Fortunately, accumulating evidence shows that aberrant miRNAs expression profiles have been associated with the development of HCC [6]. Previous study showed that miRNAs were correlated in hepatocarcinogenic effect of HCV [26]. However, different reports have the discrepancies due to samples, technical variations and analysis methods. Therefore, we conducted this meta-analysis to evaluate the clinical value of circulating miRNAs in diagnosis of HCV-HCC.

According to our results, circulating miRNAs showed high diagnostic accuracy for HCV-HCC detection, with SEN of 0.83 (95% CI, 0.79–0.87), SPE of 0.77 (95% CI, 0.71–0.82), and AUC of 0.87 (95% CI, 0.84–0.90). A significant improvement in the SEN was observed when circulating miRNAs combined with AFP than using alone (P < 0.000). Moreover, we have characterized the role of miRNA clusters as diagnostic and prognostic markers for distinction of HCV-HCC from CHC and HCV-LC subgroup.

Currently, available diagnostic or prognostic biomarkers have limited accuracy for HCC [27]. AFP is the most widely used for HCC, however, serum AFP levels are related to both HCC and benign liver diseases, such as hepatitis and cirrhosis [28, 29]. Precious studies have demonstrated that miRNAs could be served as high-precision detection of HCC biomarker [30]. In this present study, although the DOR of AFP is higher than miRNAs alone (33 versus 17), no statistical difference of OA value was observed. Similarly, He et al. found SEN and AUC-SROC of AFP for HCC were significantly less than miRNAs, while the DOR of AFP was higher than miRNAs [31]. The possible reasons for this are associated with the cut-off value of AFP, stage of HCC, and tumor size. Recent evidence indicated miRNAs had a better performance compared with AFP in detection of early-stage HCV-HCC from CHC and LC, such as miR-331-3p, miR-23b-3p, miR-19a, miR-223, miR-122, miR-199a, miR-16, miR-101–1 and miR-221 [10, 14, 21, 24]. In addition, the OA value of miRNAs combined with AFP had a significantly higher accuracy for HCV-HCC than AFP or miRNAs alone (P < 0.000). These findings together with previous results demonstrated circulating miRNAs could be used as putative diagnostic and prognostic biomarkers for HCV-HCC.

In the subgroup analyses, miRNAs from plasma had higher precision detection for HCV-HCC than that from serum. The DOR of plasm and serum miRNAs was 64 (95% CI 25–164) versus 14 (95% CI 8–23), and AUC was 0.87 (95% CI 0.84–0.90) versus 0.85 (95% CI 0.82–0.88), respectively. Previous studies reported that miRNAs concentration in plasma is higher than that in serum due to more proteins in plasma [32, 33]. However, the opposite results were also found in serum [31]. Therefore, further studies are needed to confirm application of specimen types in clinical practice. Interestingly, our study revealed differences in DOR (237 versus 12) when selecting miRNA clusters for HCV-HCC diagnosis. However, the miRNAs panel has not been definitely decided yet due to differentially expressed circulating miRNAs in HCV-HCC [13, 23]. All the above researches suggested that multiple miRNAs panel may be a promising prospect for application as a non-invasive method for HCV-HCC.

Although the results are promising, several limitations need to be addressed. First, some related studies, such as letters, editorials, case reports and conference proceedings, were not included. Second, most studies included in this meta-analysis were from Egypt, having an adverse effect on population selection bias. Third, different cut-off values were not extracted due to limited data, such as HCC characteristics and different baseline features of patients, which may result in a latent problem and high heterogeneity when interpreting the results. Fourthly, the data on special single type of miRNA were insufficient, restricting the clinical application. Therefore, the results of this study need more higher quality studies for confirmation in the future.

## Conclusions

In conclusion, miRNAs could distinguish HCV-HCC from CHC and LC. Combined application of miRNAs and AFP was more effective. In addition, the diagnostic accuracy of miRNA clusters was significantly high in HCV-HCC patients. Therefore, the results of our study strongly suggested that there is a real possibility of using circulating miRNAs as potential non-invasive biomarker of HCV-HCC.

## Supplementary Information

**Additional file 1: Table S1. **Summary diagnostic accuracy of circulating miRNAs, AFP and miRNAs combined with AFP for HCV-HCC**. Figure S1. **The quality assessment of included articles using the QUADAS-2 criteria. **Figure S2. **Forest plots of pooled sensitivity (SEN), specificity (SPE), diagnostic odds ratio (DOR), and summary receiver operating characteristic (SROC) curve of circulating miRNAs alone and combined with AFP for diagnosis of HCV-HCC among CHC patients. (a) SEN of miRNAs; (b) SPE of miRNAs; (c) DOR of miRNAs; (d) SROC curve of miRNAs; (e) SEN of miRNAs combined with AFP; (f) SPE of miRNAs combined with AFP; (g) DOR of miRNAs combined with AFP; (h) SROC curve of miRNAs combined with AFP. **Figure S3. **Forest plots of pooled sensitivity (SEN), specificity (SPE), diagnostic odds ratio (DOR), and summary receiver operating characteristic (SROC) curve of circulating miRNAs alone for diagnosis of HCV-HCC among HCV-LC patients. (a) SEN of miRNAs; (b) SPE of miRNAs; (c) DOR of miRNAs; (d) SROC curve of miRNAs.

## Data Availability

All data generated or analyzed during this study are included in this published article and its supplementary information files.

## Abbreviations

miRNAs: Circulating microRNAs
HCV: Hepatitis C virus
HCV-HCC: HCV-associated hepatocellular carcinoma
qRT-PCR: Quantitative real-time reverse transcription PCR
HCC: Hepatocellular carcinoma
LC: Liver cirrhosis
CHC: Chronic hepatitis C
SEN: Sensitivity
SPE: Specificity
AUC: Area under the curve
PLR: Positive likelihood ratios
NLR: Negative likelihood ratios
SROC: Summary receiver operating characteristic
95% CI: 95% Confidence intervals
Coef.: Coefficient
Ref.: References
DOR: Diagnostic odds ratio
