# Supplementary material for: Diagnostic accuracy of circulating microRNAs for hepatitis C virus-associated hepatocellular carcinoma: a systematic review and meta-analysis
Source: BMC Infect Dis. 2022 Apr 1;22:323. doi: 10.1186/s12879-022-07292-8 (PMC8973602; doi:10.1186/s12879-022-07292-8)

**Table S1.** Summary diagnostic accuracy of circulating miRNAs, AFP and miRNAs combined with AFP for HCV-HCC.

| Test | Number of patients | SEN (95% CI) | SPE (95% CI) | PLR (95% CI) | NLR (95% CI) | DOR (95% CI) | AUC (95% CI) |
| --- | --- | --- | --- | --- | --- | --- | --- |
| miRNAs | 6994 | 0.83 (0.79-0.87) | 0.77 (0.71-0.82) | 3.6 (2.8-4.7) | 0.21 (0.16-0.29) | 17 (10-28) | 0.87 (0.84-0.90) |
| AFP | 1871 | 0.65 (0.50-0.78) | 0.95(0.78-0.99) | 12.0 (3.0-48.3) | 0.37 (0.25-0.53) | 33 (9-125) | 0.85 (0.81-0.87) |
| miRNAs+AFP | 1276 | 0.88 (0.83-0.92) | 0.88 (0.77-0.94) | 7.1 (3.6-14.1) | 0.14 (0.25-0.53) | 51 (19-136) | 0.93 (0.90-0.95) |

HCV-HCC, HCV-associated hepatocellular carcinoma; miRNAs, MicroRNAs; AFP, alpha-fetoprotein; SEN, sensitivity; SPE, specificity; PLR, positive likelihood ratios; NLR, negative likelihood ratios; DOR, diagnostic odds ratio; AUC, area under the curve; 95% CI, 95 % confidence intervals.

**Figure S1.** The quality assessment of included articles using the QUADAS-2 criteria.

**
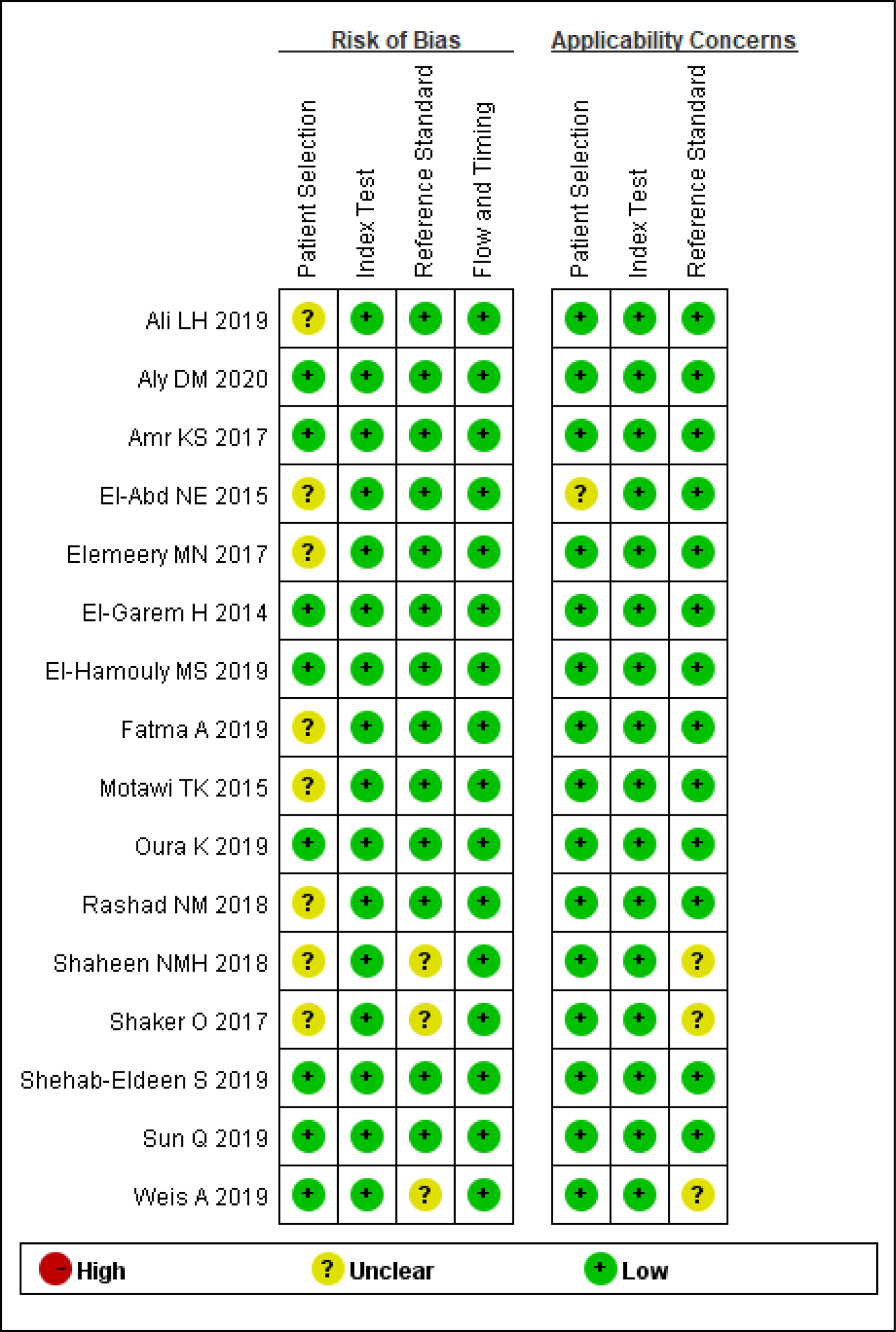
**

**Figure S2.** Forest plots of pooled sensitivity (SEN), specificity (SPE), diagnostic odds ratio (DOR), and summary receiver operating characteristic (SROC) curve of circulating miRNAs alone and combined with AFP for diagnosis of HCV-HCC among CHC patients. (a) SEN of miRNAs; (b) SPE of miRNAs; (c) DOR of miRNAs; (d) SROC curve of miRNAs; (e) SEN of miRNAs combined with AFP; (f) SPE of miRNAs combined with AFP; (g) DOR of miRNAs combined with AFP; (h) SROC curve of miRNAs combined with AFP.


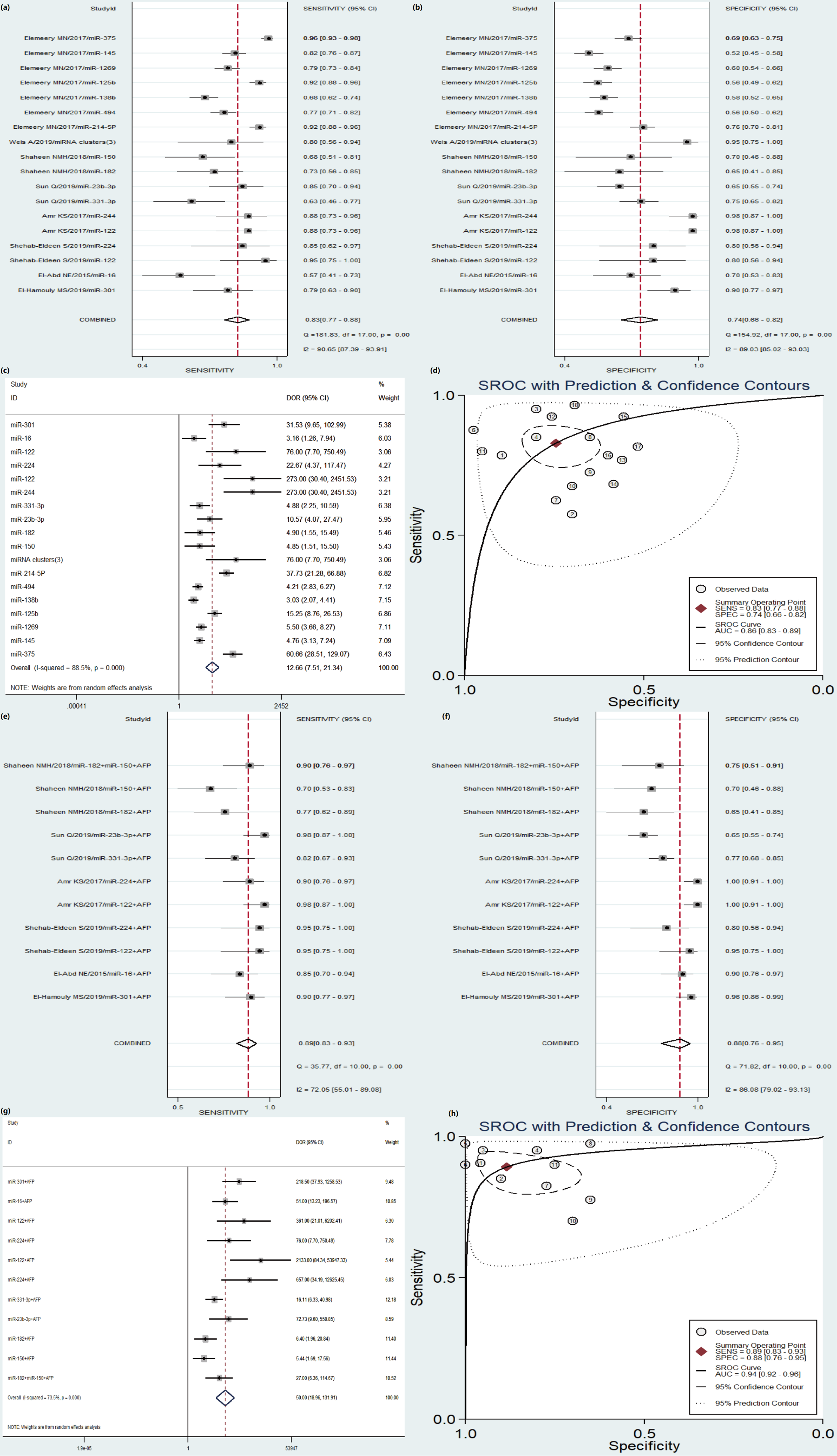


**Figure S3.** Forest plots of pooled sensitivity (SEN), specificity (SPE), diagnostic odds ratio (DOR), and summary receiver operating characteristic (SROC) curve of circulating miRNAs alone for diagnosis of HCV-HCC among HCV-LC patients. (a) SEN of miRNAs; (b) SPE of miRNAs; (c) DOR of miRNAs; (d) SROC curve of miRNAs


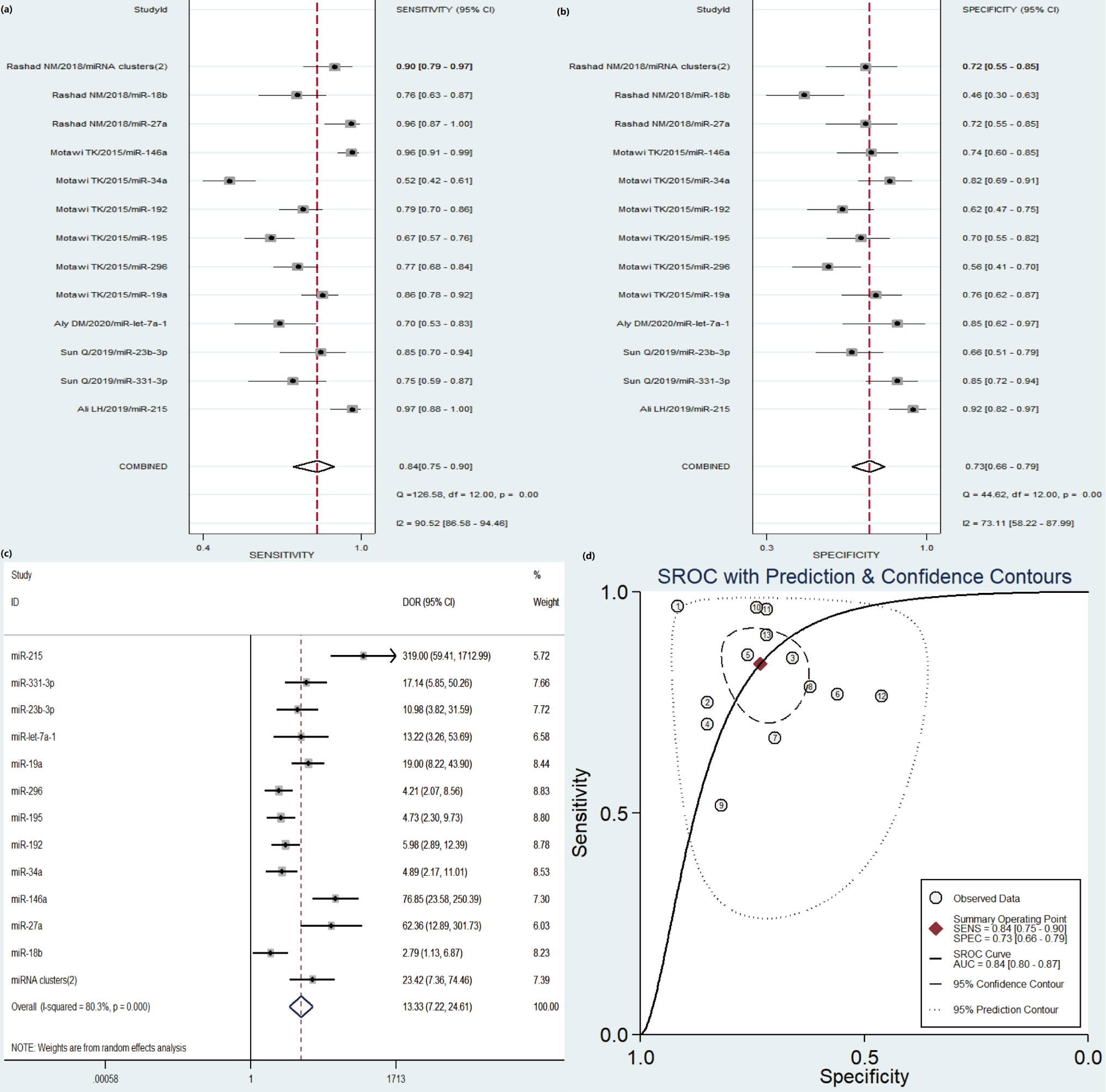

Supplement: Supplementary file 1 — Additional file 1: Table S1. Summary diagnostic accuracy of circulating miRNAs, AFP and miRNAs combined with AFP for HCV-HCC. Figure S1. The quality assessment of included articles using the QUADAS-2 criteria. Figure S2. Forest plots of pooled sensitivity (SEN), specificity (SPE), diagnostic odds ratio (DOR), and summary receiver operating characteristic (SROC) curve of circulating miRNAs alone and combined with AFP for diagnosis of HCV-HCC among CHC patients. (a) SEN of miRNAs; (b) SPE of miRNAs; (c) DOR of miRNAs; (d) SROC curve of miRNAs; (e) SEN of miRNAs combined with AFP; (f) SPE of miRNAs combined with AFP; (g) DOR of miRNAs combined with AFP; (h) SROC curve of miRNAs combined with AFP. Figure S3. Forest plots of pooled sensitivity (SEN), specificity (SPE), diagnostic odds ratio (DOR), and summary receiver operating characteristic (SROC) curve of circulating miRNAs alone for diagnosis of HCV-HCC among HCV-LC patients. (a) SEN of miRNAs; (b) SPE of miRNAs; (c) DOR of miRNAs; (d) SROC curve of miRNAs. [file 12879_2022_7292_MOESM1_ESM.docx]
